# Pulp Protection in Filling Teeth

**Published:** 1896-06

**Authors:** J. D. Fatterson

**Affiliations:** Kansas City.


ARTICLE V.

### PULP PROTECTION IN FILLING TEETH.

DR. J. D. FATTERSON, KANSAS CITY.

In considering this subject, no reference will be made to pulps which have become exposed or in a pathological condition very near exposure, but to fairly deep-seated cavities in the teeth of patients under twenty-five years of age, which are usually filled without any endeavor to prevent pulp-irritation, the operator believing that no considerable trouble will be possible, either from the presence of a filling with the property of conducting thermal changes, or from the irritant nature of filling materials in common use. In this class of cases every observant practitioner has had his attention directed to the number of severe pathological conditions arising in after years from the death of the pulp. Where there is immediate or soon occurring trouble after a filling is inserted on a nearly exposed or pathological pulp, the removal of the filling and usual treatment bring immediate cure; but where the death of the pulp comes from no continued irritation, the danger is greater. The patient's attention has not been called to the tooth till months or maybe years have elapsed, and when from an anemic or diseased systemic condition, the dead remains of the pulp-tissue overcome the healthy function of the parts, the operator, be he wise or skilful in treatment, must often fold its hands unable to abate the alarming and rapid processes of destruction.

I will attempt to make a diagnosis of these cases, and show why they are so difficult to control. When the irritation to the pulp is slight, as it must be when it penetrates through the wall of dentin, the pulp responds without noticeable pain to the physiological action. If the putrid pulp contents are papidly forced through the apical opening, the result is an immediate abscess; but in the slower process, from slight irritation, the poisonous matter infiltrates into the surrounding tissue in small quantities and intermittently, and nature, with its wonderful facility for destroying invading poisons, destroys the pathogenic bacteria and absorbs the detritus when it comes in small quantities. In this process, however, the investing tissues become weakened, and weekly or monthly the putrescent pulp matter again comes from the apical opening. Pathologists well know that an abscess voiding intermittently will seek new points of outlet, following more easily the healthy tissue than cicatrized territory. In this way each fresh invasion of pus will prejudice new territory, till irritation after irritation of the parts has placed the surrounding alveolar process and periosteal tissues, often to a distant point, in a weak and predisposed condition. There is no new power left in the tissues to withstand the attack, and at times great loss of process and teeth supervene through rapid inflammation and necrosis.

For pulp protection a variety of methods and materials have been adopted. Gutta-percha easily stands in first place, but it cannot be easily used except in deep cavities, where there is room for a good layer of cement over it, on which to condense gold. Non-cohesive gold in my practice is used for pulp protection almost to the exclusion of all other materials. Those who have been in practice many years cannot fail to notice that the old-time non-cohesive gold fillings, which forty years ago were not universally used, on removal were often found placed over pulps, which were protected only by an extremely thin layer of dentin, and in this position had not caused any uneasiness from thermal changes. If cohesive gold were used for refilling, the pulp was at once affected by hot and cold liquids and cold air, and even resulted in pulp-destruction. The philosophy of this difference in conducting power, between the two forms of gold, is not difficult to trace. In the non-cohesive filling it is in no way a cohesive mass; cannot be welded together, and when a cavity is filled with layer after layer of this gold the thermal shock is conducted along the layers and not down to the bottom of the cavity, for there is no intimate connection between the different particles of the different sheets. If you are annoyed with the effects of cold and heat in a cohesive gold filling, and will remove such filling, and without further treatment place over the pulp a pad of non-cohesive gold, as here described, I can assure you the trouble will at once disappear, and that afterward you will treat all deep-seated cavities in this manner.—*International.*

